# Sublingual Misoprostol versus Foley catheter for cervical ripening in women with preeclampsia or gestational hypertension: A randomized control trial

**DOI:** 10.18502/ijrm.v17i7.4863

**Published:** 2019-07-31

**Authors:** Sedigheh Ayati, Elahe Hasanzadeh, Leila Pourali, Mohammadtaghi Shakeri, Atiye Vatanchi

**Affiliations:** ^1^Department of Obstetrics and Gynecology, Faculty of Medicine, Mashhad University of Medical Sciences, Mashhad, Iran.; ^2^Faculty of Medicine, Mashhad University of Medical Sciences, Mashhad, Iran.; ^3^Department of Epidemiology and Biostatics, Health School, Mashhad University of Medical Sciences, Mashhad, Iran.

**Keywords:** Preeclampsia, Labor induction, Misoprostol, Pregnancy.

## Abstract

**Background:**

Delivery is the only definite cure for hypertensive disorders. Therefore, cervical ripening and labor induction are important to achieve favorable outcomes.

**Objective:**

This Randomized Control Trial (RCT) is aimed to compare the effects of sublingual misoprostol and Foley catheter in cervical ripening and labor induction among patients with preeclampsia or gestational hypertension.

**Materials and Methods:**

A total number of 144 women with preeclampsia or gestational hypertention with indication of pregnancy termination, who were referred to academic hospitals of the University of Medical Sciences in Mashhad, Iran, between March 2015 and December 2016, were randomly divided into two groups. In group one (n = 72), 25 µg of misoprostol tablet was administrated sublingually every 4 hr up to six doses. In group two (n = 72), a 16F Foley catheter was placed through the internal cervical os, inflated with 60 cc of sterile saline.

**Results:**

There were no significant differences between groups regarding the demographic characteristics, primary bishop score, and pregnancy termination indication. The cervical ripening time (primary outcome) (8.2 vs 14.2 hr, p < 0.00), induction to delivery interval (15.5 vs 19.9 hr, p < 0.00), and vaginal delivery before 24 hr (63.9% vs 40%, p = 0.03) were significantly different between the two groups. There was no significant difference between groups in view of oxytocin requirement (p = 0.12), neonatal Apgar score (p = 0.84), or neonatal intensive care unit admission (p = 78).

**Conclusion:**

This trial showed that the application of sublingual misoprostol, compared to the Foley catheter, can reduce cervical ripening period and other parameters related to the duration of vaginal delivery. This misoprostol regimen showed inconsiderable maternal complications.

## 1. Introduction

Hypertensive disorders complicate up to 10% of all pregnancies. Even though many antihypertensive agents are available to control preeclampsia, the only definite cure is delivery (1). Therefore, the induction of labor and cervical ripening are momentous procedures in order to achieve favorable outcomes and to prevent morbidity and mortality in both mother and the baby. Sublingual misoprostol and Foley catheter are two inexpensive and frequently used cervical ripening and labor-inducing methods.

During the last two decades, the rate of labor induction has increased, in so far as up to 20-30% of all labors are induced for different reasons (2). Despite the fact that these two methods are used frequently, their relative risks and benefits are unknown, and contradictory results are reported in different trials. Since the conditions of a pre-eclamptic patient are unstable, an ideal ripening method results in a relatively shorter ripening duration. Moreover, leading to a fast vaginal delivery without increasing risk to the fetus or the rate of urgent cesarean delivery seems to be a necessary feature.

This study is aimed to evaluate and compare the effect of sublingual misoprostol and Foley catheter in cervical ripening among pregnant women with preeclampsia or gestational hypertension.

## 2. Materials and Methods

**Figure 1:**
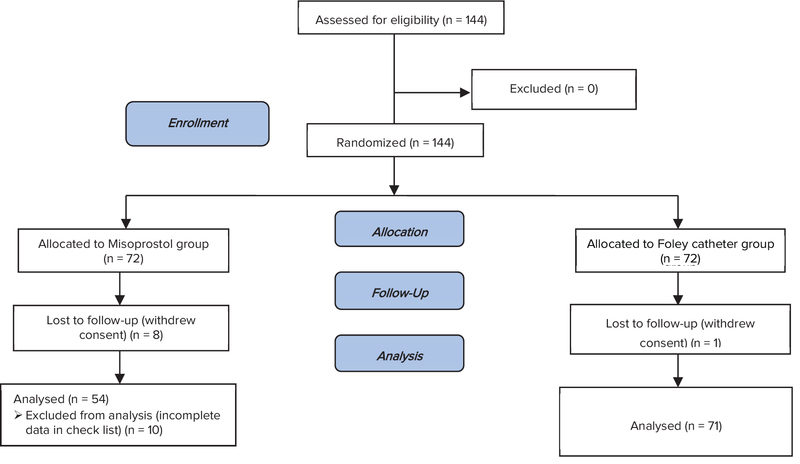
Consort flow diagramfor participants involved in the trial.

This randomized clinical trial was conducted among 144 women with preeclampsia or gestational hypertention with indication of pregnancy termination in Ghaem and Imam Reza Hospitals, Mashhad University of Medical Sciences, Iran, between March 2015 and December 2016. Patients were randomly allocated to sublingual misoprostol (n = 72) or Foley catheter group (n = 72) using the sealed envelope system. During the study, 18 patients in the misoprostol group and 1 patient in Foley catheter group were excluded from the trial (Figure 1).

The inclusion criteria were as follows: preeclampsia or gestational hypertension with indication of pregnancy termination, singleton pregnancies, gestational age of > 26 wk, cephalic presentation, unfavorable cervix (bishop score < 6), the absence of active labor, normal fetal heart rate (FHR), and the absence of previous cesarean delivery or uterine scar or macrosomia or Placenta Previa. We have excluded individuals with hypersensitivity to prostaglandins and those who could not continue the study.

In the misoprostol group, 25 µg misoprostol (1/4 of a 100 µg misoprostol tablet; Cytotec, Searl & Co, England) was administered sublingually every 4 hr up to a maximum of six doses, if needed. In case of a non-reassuring FHR or in the presence of three or more contractions in 10 min, a subsequent misoprostol dose was withheld. Moreover, if augmentation was required, a low-dose Oxytocin regimen infusion was used (2 mlU/min initially, drops were increased 2 mlU/min each 20 min if needed).

In Foley Catheter group, a 16 French size silicone Foley catheter was placed in the cervix through the internal orifice of the cervix (internal os) using a vaginal speculum under aseptic condition, then it was inflated with 60 cc of sterile saline. The external end of the Foley catheter was taped to the thigh without traction. The catheter was expelled spontaneously or by the physician if the bishop score remained less than 6 after 24 hr of insertion. After catheter expulsion, a low-dose Oxytocin regimen was used if contractions were less than 4 in 10 min.

During the intervention, FHR was monitored continuously. A gynecology resident examined patients every 2 hr. If uterine tachysystole (6 or more contractions in 10 min) occurred, the patient was positioned laterally; a 500 cc Ringer lactate infusion and a 10 Lit/m oxygen therapy were applied.

In both groups, cesarean section was performed in case of cervical dilatation arrest (if no cervical dilatation happened during active phase in 2 hr), fetal descent arrest (if no fetal descent happened during the second stage of delivery in 1 hr), or fetal distress (in case of late, prolonged, or significant variable decelerations in the cardiotocography). All patients were followed up until 24 hr after the delivery in order to check any possible side effect.

Our primary outcome was the cervical ripening duration that defined as the period of the time between the commencement of the intervention and the first vaginal exam in which the patient's bishop score was more than 6. Secondary outcomes were oxytocin requirement, cesarean delivery, induction to delivery interval, the vaginal delivery rate in 24 hr, maternal side effects (post-delivery hemorrhage, fever within 24 hr after delivery, diarrhea, and tachysystole), neonatal Apgar score, and neonatal intensive care unit (NICU) admission.

According to the data from the trial by Vahid and colleagues, our trial sample size with 95% confidence interval and 80% power was calculated in at least 36 subjects in each group (3). We doubled it to 72 in order to increase accuracy.

### Ethical consideration

This trial was approved by the ethics committee of the Mashhad University of Medical Sciences (no: IR.MUMS.REC.1394.498) and by the Iranian Registry of Clinical Trials (IRCT2017010731725N1). All participants provided oral and written informed consent. Due to the nature of the interventions, blinding was not possible.

### Statistical analysis

Statistical analyses were done by SPSS software; (Statistical Package for the Social Science, version 16.0, SPSS Inc., Chicago, IL, USA). The *t*-test (for independent and dependent samples) was used for the comparison of quantitative data. Nonparametric methods were used in cases where data were not normally distributed. The chi-squared test was used to compare the studied variables. We considered p < 0.05 as significant.

## 3. Results

125 women with the mean age of 26.1 ± 4.5 yr, ranged between 17-36 yr old were enrolled. Misoprostol and Foley catheter groups were similar in the view of demographic characteristics including age, gestational age, parity, and primary Bishop score (p > 0.05; Table I). The majority of patients in both groups were nulliparous and had Bishop score less than three at recruitment. The mean administrated dose in the misoprostol group was 2.3 **** ***±*** ****0.7 and most of the patients received 2–3 doses of misoprostol (n: 45, 83.3%).

The cervical ripening time (primary outcome) and induction to vaginal delivery interval were significantly shorter in the misoprostol group compared to the Foley catheter group (p < 0.001; Table II). Vaginal birth within 24 hr was significantly more common in women in the misoprostol group than in the Foley catheter group (63.9% vs 40.0%, p = 0.03). Oxytocin augmentation requirement was higher in the Foley catheter group; however, this difference did not reach statistical significance (p = 0.12). Among cases who had to undergo cesarean section, there was no statistical difference in the indications for cesarean section between two groups (p = 0.45).

Generally, maternal complications were more common in the Foley catheter group than the misoprostol group. One woman (1.4%) in the Foley group had postpartum bleeding due to atonia; three women in the Foley catheter group (4.2%) and one woman in the misoprostol group (1.8%) had fever within 24 hours after the delivery. One woman (1.8%) in the misoprostol group experienced uterine tachysystole.

There was no significant difference between groups in view of neonatal fifth minute Apgar score, the rate of NICU admission, and NICU admission due to prematurity (p = 0.84, p = 0.78 p = 0.67, respectively).

**Table 1:** Baseline clinical characteristics of misoprostol and Foley catheter group


**Variable**	**Misoprostol group (n: 54)**	**Foley catheter group (n: 71)**	**Total (n: 125)**	**P-value**
Maternal age (yr)*	26.1 ± 4.3	26.1 ± 4.5	26.1 ± 4.4	0.97
Gestational age (wk)*	35.1 ± 2.4	34.7 ± 2.5	34.9 ± 2.5	0.89
Primary Bishop score:**				
0–2	34 (63.0)	48 (67.6)	82 (65.6)	
3–6	20 (37.0)	23 (32.4)	43 (34.3)	<brow>-2</erow> 0.58
Type of parity:**				
Nulliparous	31 (57.4)	49 (69.0)	80 (64.0)	
Multiparous	23 (42.6)	22 (31.0)	45 (36.0)	<brow>-2</erow> 0.18
Pregnancy termination Indication:**				
Gestational hypertension	13 (24.0)	18 (25.0)	31 (24.8)	
Preeclampsia	37 (68.5)	49 (69)	86 (68.8)	
Eclampsia	4 (7.4)	4 (5.6)	8 (6.4)	<brow>-3</erow> 0.91
*Data presented as mean ± SD				
**Data presented as n (%)				
The *t*-test was used to analyze continuous data, and Chi-Square and Mann–Whitney test were used for nonparametric data analysis				

**Table 2:** Comparison of study outcomes between Misoprostol and Foley catheter groups


**Variable**	**Misoprostol group (n:54)**	**Foley catheter group (n:71)**	**P-value**
Cervical ripening time (hr)*	8.2 ± 3.8	14.2 ± 4.2	< 0.001
Induction to vaginal delivery interval (min)*	15.6 ± 7.5	20.0 ± 7.0	< 0.001
Vaginal birth within 24 hr**	23 (63.9)	18 (40.0)	0.03
Oxytocin requirement**	17 (31.5)	32 (45.5)	0.12
Vaginal delivery**	36 (66.7)	45 (63.4)	0.70
Cesarean section due to fetal distress**	12 (66.7)	20 (76.9)	0.45
Fifth minute apgar over 8**	41 (82)	56 (80)	0.84
NICU admission**	14 (25.9)	20 (28.2)	0.78
NICU admission due to prematurity**	11 (78.6)	16 (84.2)	0.67
*Data presented as mean ± SD			
**Data presented as n (%)			
The *t*-test was used to analyze continuous data and Chi-Square test was used for nonparametric data analysis			

## 4. Discussion

The results of this study showed that sublingual misoprostol comparing to Foley catheter significantly reduces cervical ripening time. In two studies by Adeniji and colleagues and a study by Aduloju and co-workers, the same result was reported (4-6).

In this trial, the mean induction-to-delivery interval was significantly shorter in the misoprostol group than in the Foley catheter group. This was similarly reported in some previous studies (7-11). In addition, a nonsignificant shorter induction-to-delivery interval in misoprostol group was reported by Barrilleaux *et al.* and Fox and co-workers (12, 13). On the other side, Prager and colleagues reported a significantly shorter induction-to-delivery interval in the catheter group compared to the misoprostol group, which is in contrary to our findings (14). In Prager study, the catheter was under traction and misoprostol was administered vaginally; however, in our study, we did not put catheter under traction and we administered misoprostol sublingually. Sublingual administration of misoprostol can lead to a quicker onset of action and a higher bioavailability compared to vaginal rout.

In this research, sublingual misoprostol led to a higher vaginal delivery rate within 24 hours. Chen and coworkers have reported the same result in their meta-analysis (15). Furthermore, some other studies reported a higher vaginal delivery rate within 12 hours in the misoprostol group compared to the Foley catheter group (10, 16). In this trial, an insignificant fewer number of women in the misoprostol group required oxytocin augmentation rather than those in Foley catheter group. Barrilleaux and colleagues also declare the same result in their trial (12). However, some researchers reported a significantly higher oxytocin requirement in Foley catheter group (6, 16-18).

This study demonstrated no significant difference in the mode of delivery between the two groups, which was also reported in similar previous studies (5, 6, 9, 19). Contrary to our study, Vahid and colleagues and Noor *et al.* reported a significantly higher rate of vaginal delivery in the misoprostol group rather than the Foley catheter group (3, 11). Vaginal administration of misoprostol and term gestational age among their patients can be an explanation for this contradiction. Since the success rate of labor induction can be higher in multiparous women rather than nulliparous ones, multiparous status in majority of Vahid`s women might be another reason for this incompatibility. Moreover, our study was limited to subjects with preeclampsia and gestational hypertension that can be a cesarean indication by itself and may lead to a higher rate of cesarean delivery compared to non-preeclamptic patients (20).

Our trial showed few maternal complications in each group. Three cases in Foley catheter group experienced fever within 24 hr after delivery that can be related to chorioamnionitis. In the catheter group, 60% of the patients experienced a prolonged induction-to-delivery interval (over 24 hr), which can be a risk factor for chorioamnionitis. On the other hand, only one case of fever within 24 hr after the delivery was reported in the misoprostol group that might have happened as a misoprostol side effect.

Among cases that underwent cesarean section in both groups, most cesarean sections were performed due to fetal distress; however, cesarean indications were not significantly different between the two groups. Therefore, we cannot relate fetal distress to any of these labor-inducing methods. Moreover, both groups were similar in the view of the first- and fifth-minute neonatal Apgar score and rate of NICU admission or NICU admission indication. Some other studies reported the same results (3, 6, 17, 18).

Sublingual administration of misoprostol in cervical ripening and labor induction is the main strength of this study and only a few trials have studied this method before, especially on high-risk patients. In a recent study, a mean ripening time was obtained in both misoprostol and Foley catheter groups that can practically be useful; this variable has not been defined exactly in former similar studies. Since no 25 µg misoprostol tablet exists, we manually divided each 100 µg tablet into four equal pieces using a pill cutter that could influence the exact dosage of misoprostol. However, it does not disprove the results of the current trial.

In view of the fact that few complications for sublingual misoprostol were reported in this trial, it seems reasonable to conduct studies in future in order to compare sublingual misoprostol to buccal or other new routs of misoprostol administration.

##  Conflict of Interest

The authors declare there is no conflict of interests regarding the publication of this manuscript.
